# Cannabis use and glutamate across the psychosis spectrum: in vivo evidence from 7T proton magnetic resonance spectroscopy

**DOI:** 10.1038/s41380-026-03705-1

**Published:** 2026-06-27

**Authors:** David R. Roalf, Tyler M. Moore, Jacquelyn Stifelman, Maggie K. Pecsok, Ally Atkins, Erica B. Baller, Monica E. Calkins, Mariella De Biasi, Christian G. Kohler, Christina Mastracchio, Arianna Mordy, Heather Robinson, Ravinder Reddy, Ravi Prakash Reddy Nanga, Kosha Ruparel, Sage Rush-Goebel, Daniel H. Wolf, Ruben C. Gur, Raquel E. Gur, J. Cobb Scott

**Affiliations:** 1 Department of Psychiatry, University of Pennsylvania Perelman School of Medicine, Philadelphia, PA USA; 2 Lifespan Brain Institute, Department of Child and Adolescent Psychiatry and Behavioral Sciences, Children’s Hospital of Philadelphia, Philadelphia, PA USA; 3 Pharmacology Graduate Group, Perelman School of Medicine, University of Pennsylvania, Philadelphia, PA USA; 4 Department of Neuroscience, Perelman School of Medicine, University of Pennsylvania, Philadelphia, PA USA; 5 Department of Psychology, University of California Los Angeles, Los Angeles, CA USA; 6 Department of Psychological Sciences, University of Connecticut, Storrs, CT USA; 7 Center for Advanced Metabolic Imaging in Precision Medicine (CAMIPM), Perelman School of Medicine, University of Pennsylvania, Philadelphia, PA USA; 8 VISN4 Mental Illness Research, Education, and Clinical Center at the Corporal Michael J. Crescenz VA Medical Center, Philadelphia, PA USA

**Keywords:** Neuroscience, Schizophrenia

## Abstract

Cannabis use is linked to elevated psychosis risk, yet the neurobiological mechanisms that couple use to symptom expression remain unclear. Because glutamatergic dysregulation has been implicated in both cannabis effects and psychosis vulnerability, we examined whether brain glutamate relates to dimensional psychosis symptoms as a function of cannabis use across the psychosis spectrum. Seventy-nine participants—typically developing controls, clinical high-risk individuals, and patients with psychosis—completed dimensional clinical assessments, detailed cannabis use surveys, urine toxicology, and ultra-high-field 7T magnetic resonance spectroscopy (^1^HMRS) of the anterior cingulate cortex (ACC). Linear models assessed the main and interactive effects of ACC glutamate and cannabis use on psychopathology symptoms. Self-reported cannabis use showed good concordance with urine toxicology, with strongest agreement among frequent users. Both lower ACC glutamate and higher cannabis use were independently associated with positive and negative psychosis symptoms. Notably, lower glutamate levels were associated with higher positive symptoms in cannabis users but not cannabis non-users. Exploratory analyses suggested interactions for depressive and manic symptoms, indicating that glutamatergic abnormalities may amplify the overall severity of cannabis-related symptoms. Sensitivity analyses revealed lower ACC glutamate in psychosis patients—especially cannabis users—highlighting diagnostic group differences and reinforcing the link between cannabis exposure and glutamatergic dysfunction. These findings implicate ACC glutamatergic dysfunction as a transdiagnostic correlate of symptom burden, particularly in those with psychosis who are cannabis users. Glutamate-targeted interventions and longitudinal designs will be needed to examine causal pathways linking cannabis exposure to psychosis-relevant outcomes.

## Introduction

Recent changes in cannabis laws and the rise in nonmedical cannabis use have intensified concerns about long-term effects of cannabis on mental health, particularly among youth. While cannabis use has been implicated in a range of psychiatric outcomes [1], accumulating evidence highlights its association with dimensional psychopathology, including positive and negative psychosis-spectrum symptoms [2] and mood disturbances [3]. Epidemiological studies show that frequent use—especially of high-potency cannabis—and earlier age of initiation are linked to greater risk for adverse outcomes [4–7], with a dose-response relationship, particularly in psychosis (PSY) [8, 9]. Cannabis use during adolescence has been associated not only with onset of psychotic disorders in early adulthood but also with elevated subclinical symptoms and poorer functional outcomes across multiple domains [10, 11]. In clinical high-risk for psychosis (CHR) samples [12–14], cannabis use is associated with worsened subthreshold symptoms, and among those with established schizophrenia, it may exacerbate symptom severity and increase relapse risk [15–17]. These effects may be partly mediated by cannabis-induced disruption of the glutamatergic system [18, 19], a key neurochemical pathway implicated in mood, psychosis, and neurodevelopmental disorders. Both cannabis use [18, 19] and psychiatric disorders [20–24] have been independently associated with glutamate dysregulation, yet the mechanistic interplay remains poorly understood. Here we begin to address this gap by integrating detailed self-reported cannabis use, urine drug screening, and ultra–high field 7T proton magnetic resonance spectroscopy (¹HMRS) of the ACC to examine glutamate (Glu) alterations associated with cannabis use across a transdiagnostic sample.

The primary psychoactive component in cannabis, Δ9-tetrahydrocannabinol (THC), modulates neurometabolic and neurotransmitter systems—most notably dopamine and glutamate [19, 25], both central to psychosis. Sustained cannabis use reduces glutamate signaling *via* Cannabinoid receptor 1 (CB1R) activation, and CB1R agonists induce psychosis-like behaviors [26]. Glutamate hypofunction [20, 21] contributes to dopamine dysregulation in schizophrenia, and recent studies have documented glutamatergic metabolite alterations in individuals with or at risk for schizophrenia [20, 22–24] and depression [27]. However, systematic in vivo investigations of glutamatergic alterations associated with cannabis use in humans using ultra-high field MRI are limited, hindering efforts to clarify how cannabis may heighten psychopathological vulnerability and symptoms.

Glutamatergic alterations are central to the pathophysiology of psychosis [28], as evidenced by a growing literature [29, 30], including data from animal models [31–35], postmortem studies [36–39], genetic analyses [40–44], peripheral biomarkers [45], and in vivo ^1^HMRS [20, 23, 46]. Yet the ^1^HMRS literature is characterized by considerable heterogeneity, partly due to variation in region-of-interest placement. Meta-analyses of 3T ^1^HMRS show that when Glu alone is examined, levels tend to be lower in psychosis in medial prefrontal cortex, frontal white matter, medial temporal lobe, and thalamus, while elevated in cerebellum, and basal ganglia [20, 23, 46]. Mega-analyses [20], 7T GluCEST investigations [22, 24], and 7T ^1^HMRS studies [23] further demonstrate regionally lower Glu in psychosis (but see [47]). Quantification methodology is also a key source of variability. At 3T, spectral overlap between Glu and glutamine (Gln) often necessitates a composite measure (Glx), which may obscure opposing effects on Glu vs. Gln. Indeed, when Gln or Glx levels were reported rather than Glu alone, cases showed consistently higher levels than controls, with the strength of this elevation depending on patient subgroup and brain region [46]. Ultra-high field 7T ^1^HMRS offers over 50% improvement in signal variability for Glu and substantially improved reliability of coupled metabolites [48, 49]. Medication effects contribute additional inconsistency, as antipsychotics may lower Glx [47, 50], yet meta-analyses of ^1^HMRS findings in neuroleptic-naïve patients found few consistent Glu differences [51]. Finally, illness stage and diagnostic heterogeneity may further confound interpretations. ^1^HMRS findings in CHR are particularly inconsistent, with individual studies reporting lower, higher, and null Glu differences across brain regions and cohorts [29, 52–61]. Taken together, these findings suggest that Glu alterations track with illness progression across the psychosis spectrum [29, 52–54, 56, 58, 62], but methodological and clinical factors have obscured the consistency of this relationship. Cannabis use patterns, which are prevalent in this population but rarely assessed systematically, may represent an additional and underappreciated source of heterogeneity, motivating the present study’s integration of detailed cannabis phenotyping and 7T ^1^HMRS measurement.

Few studies directly examined cannabis–glutamate associations across clinical dimensions of the psychosis spectrum, and existing work has been limited to 3T MRI. Early ^1^HMRS work found that patients with early psychosis who used cannabis exhibited lower prefrontal Glu concentrations than both non‐using psychosis patients and healthy controls, along with a steeper age-related Glu decline, suggesting that cannabis may exacerbate glutamatergic dysfunction and influence disease progression [63]. Another 3T ^1^HMRS study in early psychosis observed limited differences in Glx between cannabis users and nonusers but identified associations between regional gray matter volume (caudate, hippocampus, cortex) and caudate Glx in cannabis users, implying that heavy cannabis use may alter structure–neurochemical connectivity in CB1R‐rich regions [64]. In healthy individuals, acute THC induced greater Glx surges in those who developed transient psychotic-like symptoms, particularly among participants with lower baseline Glx and greater prior cannabis exposure [65]. These findings collectively suggest that baseline glutamatergic state and cannabis responsiveness may interact to influence psychosis risk and expression. However, prior studies have been limited to 3T ^1^HMRS, where sensitivity to glutamate is lower, and they have only measured cannabis–glutamate associations in categorical clinical groupings.

In summary, converging epidemiological, preclinical, and neuroimaging evidence implicates glutamate as a key modulator of the cannabis–psychosis relationship. By integrating detailed cannabis use phenotyping, 7T ^1^HMRS, and dimensional clinical assessments across typically developing, CHR, and PSY individuals, our study aims to elucidate how cannabis use is associated with glutamatergic levels across the psychosis spectrum. Consistent with RDoC and NIMH initiatives, we assess symptoms dimensionally rather than relying solely on categorical diagnoses, providing greater sensitivity to subthreshold variation [66, 67], facilitating transdiagnostic comparisons, and improving power to detect brain–behavior associations. Understanding this interplay may identify neurobiological and clinical markers of risk to inform targeted prevention and intervention strategies.

## Methods

### Participants

To capture a broad range of psychopathology, we studied both healthy individuals and those with subthreshold and threshold psychosis symptoms. The study sample (*N* = 79, ages 14–32, 35 females) included a typically-developing (TD) group (no DSM diagnoses or subthreshold psychosis symptomatology, no history of psychotropic medication use; *N* = 31) and a clinical group composed of individuals classified by experienced clinician scientists (CGK, MEC) as having psychosis spectrum disorders (*N* = 48), including 27 with CHR and 21 with schizophrenia spectrum and other psychotic disorders (PSY; *N* = 21). Although our primary analyses treat psychopathology as a continuous, transdiagnostic construct, demographic and clinical characteristics are displayed by categorical diagnosis, to facilitate evaluation of sample representativeness and provide context for dimensional analyses presented below. Complete demographic and clinical information are presented in Table 1, stratified by cannabis user status and clinical diagnosis. In addition, sensitivity analyses are conducted by diagnostic group (Supplemental Results). Brief cognitive (Montreal Cognitive Assessment (MoCA [68])) and functional assessments (Global Assessment of Functioning (GAF [69])) were also administered. The MoCA provides a practical cognitive screener that has demonstrated utility in detecting cognitive impairment across the psychosis spectrum [70–72]. Participants provided informed consent/assent and the study procedures were approved by the Institutional Review Boards at Penn.

**Table 1:** Participant demographics and clinical characteristics.

	N	Age (years)	% Female	SOPS Positive	SOPS Negative	SOPS Disorganized	SOPS Generalized	GAF	MoCA
**TD**									
Cannabis User									
No	16	20.7 ± 2.7	63%	1.3 ± 1.8	1.1 ± 1.0	0.4 ± 0.6	0.7 ± 0.9	87.4 ± 4.5	26.5 ± 2.8
Yes	15	22.8 ± 3.0	27%	1.7 ± 1.8	1.5 ± 1.8	0.3 ± 0.6	1.5 ± 2.0	81.2 ± 13.7	27.7 ± 1.6
**CHR**									
Cannabis User									
No	14	20.8 ± 3.6	50%	8.1 ± 4.7	7.6 ± 6.0	4.1 ± 2.5	3.7 ± 3.4	56.9 ± 8.0	24.8 ± 2.9
Yes	13	22.6 ± 2.8	31%	6.8 ± 3.1	7.6 ± 5.5	4.0 ± 3.0	3.2 ± 2.5	61.0 ± 12.2	27.0 ± 2.7
**PSY**									
Cannabis User									
No	5	26.8 ± 4.1	60%	14.0 ± 6.1	9.4 ± 5.6	5.4 ± 1.1	5.6 ± 3.6	47.4 ± 4.0	22.0 ± 3.9
Yes	16	24.8 ± 3.5	44%	19.7 ± 6.0	12.0 ± 2.7	7.6 ± 4.2	8.5 ± 6.0	42.4 ± 9.0	25.6 ± 2.4

This table summarizes demographic and clinical characteristics of the study sample. Demographic variables include age, sex distribution, and relevant clinical status or group designation. Mean (+/− s.d.) positive, negative, disorganized and generalized SIPS, GAF and MoCA scores are shown.

Participant demographics (mean ± s.d.) stratified by clinical diagnosis (TD, CHR, PSY) and cannabis use. *TD* typically developing, *CHR* clinical high risk, *PSY* psychosis. *SOPS* scale of prodromal symptoms, *GAF* global assessment of functioning, *MoCA* montreal cognitive assessment.

### Clinical assessment

All participants received detailed clinical assessments, including a semi-structured diagnostic interview to assess a broad spectrum of psychosis-relevant experiences, psychopathology, treatment history, and medications. [73] Participants received diagnostic assessments by highly-trained assessors using a computerized version of an adapted *Schedule for Affective Disorders and SZ for School Age Children–Present and Lifetime Version (K-SADS-4.0)* [73], which includes semi-structured assessments of mood, ADHD and substance use disorders. Psychosis spectrum symptoms were assessed using the *Structured Interview for Prodromal Syndromes (SIPS, v. 4.0)*, in which selected modules of the Structured Clinical Interview for DSM-IV (SCID-IV) [74] are integrated to facilitate differential diagnosis. The *Scale of Prodromal Symptoms (SOPS)* [75], embedded within the SIPS [76], describes and dimensionally rates the severity of positive, negative, disorganized, and general symptoms occurring within the past 6 months. CHR was defined as the presence of at least one positive symptom rated 3–5, or at least two negative and/or disorganized symptoms rated 3–6. Psychosis (PSY) patients are individuals with a DSM schizophrenia spectrum disorder diagnosis (e.g., SZ). Self-reported alcohol and nicotine use were recorded. See Supplemental Methods for more details on the clinical assessment.

### Estimation of correlated traits clinical factor scores

Clinical factor scores for participants enrolled in the current study were derived using a framework that incorporated longitudinal computerized assessments (e.g. GOASSESS [77, 78] and CAPA [79]) used in our laboratory since the landmark Philadelphia Neurodevelopmental Cohort study [80–82] to inform a dimensional factor analysis. Because the same instruments were not used across historical time points, historical data were harmonized across longitudinal visits by using only items that overlapped between GOASSESS and CAPA interviews. These included the screen items from depression, mania, and ADHD sections of the GOASSESS/CAPA, along with the PRIME [83, 84] screen and a subset of SIPS items. Using this harmonized framework, dimensional clinical scores (correlated trait factor scores) were generated for each participant based on CAPA data collected (described in the Clinical Assessment section). Dimensional clinical factor scores are shown, by diagnostic group, in Supplementary Table 2.

### Cannabis use

Cannabis use was measured using a computerized version of the Minnesota Center for Twin and Family Research self-report substance use assessment [85], which was privately self-administered. The measure assessed lifetime use of several substances; for cannabis and alcohol, additional questions queried age at first use and frequency of past year use. To obtain more detailed data regarding patterns of cannabis use, several questions from the *DFAQ-CU* (*Daily Sessions, Frequency, Age of Onset, and Quantity of Cannabis Use Inventory*) [86] were also administered. This measure assessed individual methods of cannabis use, with visual depictions to indicate quantities of cannabis across forms (e.g., joint, flower). Summary measures were generated for frequency, age of onset, and quantity of cannabis use across use methods, including increasingly popular methods (e.g., edibles, concentrates). The DFAQ-CU shows convergent, predictive, and discriminant validity, including high expected correlations (>0.7) with other frequency measures (e.g., timeline follow-back), moderate to high correlations with cannabis use disorder symptoms and cannabis-related problems, and low correlations with alcohol use problems [86, 87]. Consistent with prior research [88, 89], we classified individuals who reported use more than once per month over the past year as *cannabis users*; for exploratory analyses we also classified individuals who reported using cannabis on a daily or nearly daily basis for the past 12 months as *frequent cannabis users* if they reported using cannabis on a daily or nearly daily basis for the past 12 months. We also administered sections of the *Cannabis Experiences Questionnaire (CEQ)* that assess motivations for cannabis use and positive and negative experiences associated with use, consistent with prior studies in early psychosis [90].

### Urine drug screen for cannabis

In line with expert consensus recommendations [91], we used a urine drug test to identify presence/absence of cannabis use. All urine samples (90 ml) were collected using Redwood Toxicology iCUPs™, which detect one or more of the primary metabolites of THC via binary immunoassay (positive or negative; detection threshold: 50 ng/mL THC-COOH). Cannabis metabolites are typically detected for up to 3 days after a single use in occasional users and considerably longer in frequent users, though detection windows vary with use patterns and individual factors. Urine drug screening occurred prior to the 7T MRI session. If a participant reported cannabis use on the day of the MRI, or if acute intoxication was suspected, the study visit was rescheduled.

### 7T ^1^HMRS data acquisition

All ^1^HMRS data were acquired on a whole‐body 7 Tesla Terra MRI system (Siemens, Erlangen, Germany) equipped with a single-channel volume transmit and 32‐channel receive proton phased array head radiofrequency coil (Nova Medical, Wilmington, MA, USA). Prior to spectroscopy, high‐resolution structural images were obtained using a magnetization‐prepared rapid acquisition gradient echo (MP2RAGE) sequence (TE = 2.52 ms, TR = 5000 ms, TI1 = 700 ms, TI2 = 2500 ms, flip angle 1 = 7°, flip angle 2 = 5°, voxel size = 0.8 mm^3^). These anatomical images served both for voxel placement and for later tissue‐segmentation corrections.

### Voxel localization and shimming

^1^HMRS voxels (10 × 40 × 15 mm³) were manually placed in anterior cingulate cortex, using the mid‐sagittal anatomical image as reference. Care was taken to align all voxel faces orthogonal to the cortical surface and to minimize inclusion of cerebrospinal fluid (CSF)‐rich sulcal regions. First‐ and second‐order B₀ shimming was performed manually over each voxel to achieve a full‐width at half‐maximum (FWHM) linewidth of ≤ 20 Hz for the unsuppressed water peak within the voxel.

The ACC was selected as the region of interest for several reasons. First, the ACC is one of the most commonly studied regions in the ^1^HMRS psychosis literature [23], facilitating comparison with prior work. Second, the ACC is a CB1 receptor-rich cortical region that shows volumetric alterations in cannabis users with schizophrenia [92], making it particularly relevant for examining cannabis–glutamate interactions. Third, practical considerations at ultra-high field favor the ACC, which provides reliable spectral quality at 7T, and the single-voxel design was chosen to maximize measurement quality rather than distributing limited scan time across multiple regions.

### Spectroscopy sequence

Spectra were acquired using a point‐resolved spectroscopy (PRESS) sequence optimized for 7T. Sequence parameters were as follows: TE = 23 ms, TR = 3000 ms, number of averages (NA) = 64 (total acquisition time ≈ 3 min 24 s per voxel). Water reference scan was obtained from the same voxel with identical parameters except that the NA was 8 and water saturation was off (See Supplemental Methods for additional details).

### Data processing and quantification

**Fig. 1:**
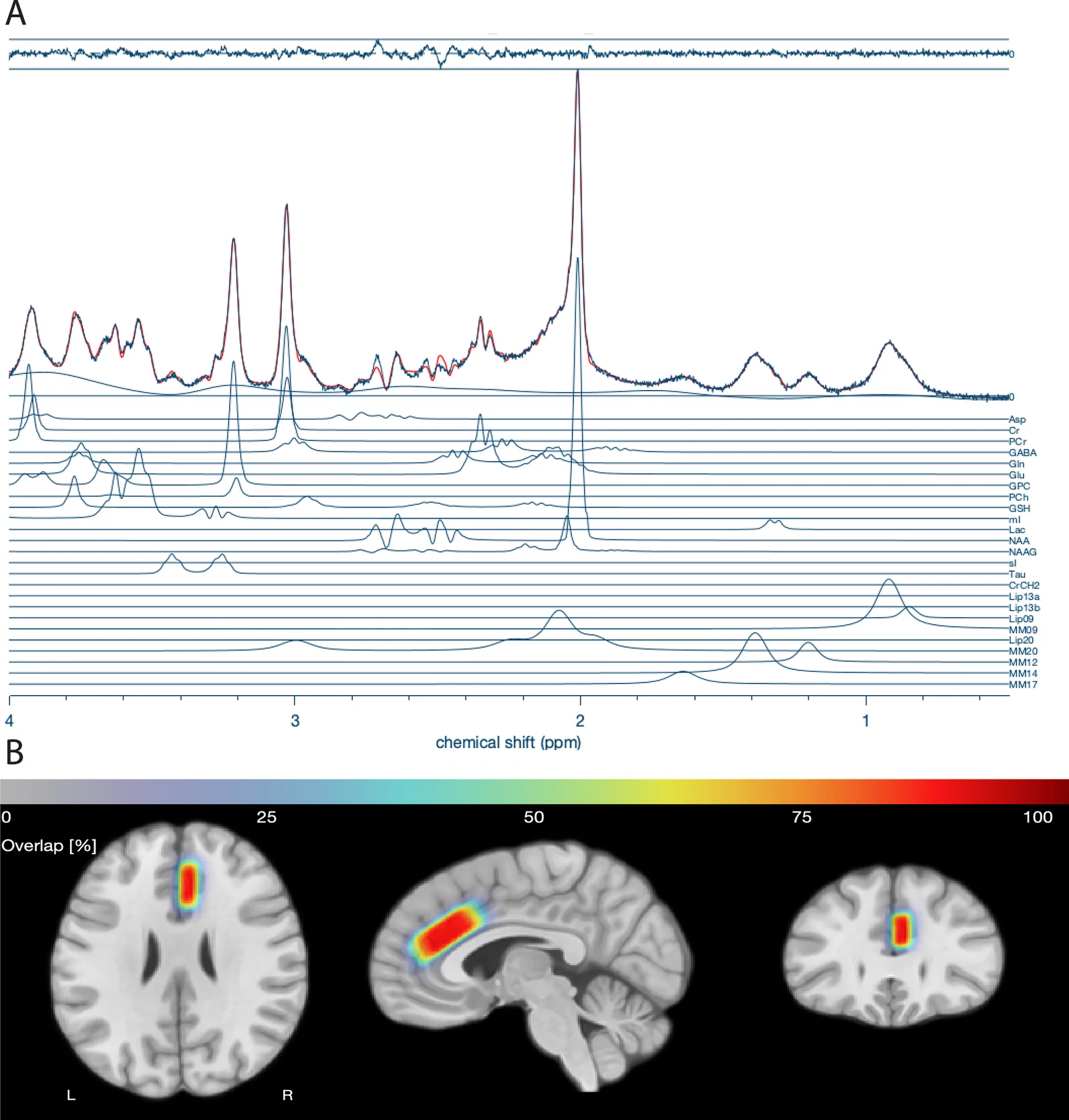
Individual metabolite quantification and voxel overlap in single-voxel spectroscopy (SVS) ¹HMRS. **A** Displays individual metabolite estimates obtained using the Osprey analysis pipeline. Each line represents the concentration of a specific metabolite quantified within the spectroscopy voxel for a representative participant. Key metabolites include glutamate (Glu), γ-aminobutyric acid (GABA), N-acetylaspartate (NAA), creatine (Cr), choline (Cho), myo-inositol (mI), and others derived from short-TE PRESS acquisitions. **B** Shows a voxel overlap heatmap across all participants, generated by co-registering individual SVS voxel placements to a common template space. Warmer colors indicate regions where greater numbers of participants’ voxels overlap, demonstrating the consistency of voxel localization across the sample and providing a measure of spatial coverage and reproducibility of the ^1^HMRS acquisition.

All spectroscopy data were exported in RDA format, which were coil-combined and averaged at the scanner. All spectra were processed, modeled, and quantified using Osprey (v2.9.0; Osprey Project, Johns Hopkins University) [93] running in MATLAB (R2023b; MathWorks, Natick, MA, USA). Spectral fitting was performed using the LCModel package (v6.3-1 N) implemented in Osprey. Prior to fitting, imported raw spectra were processed through the ‘OspreyProcess’ module, which includes eddy-current correction, frequency and phase alignment, water removal, frequency referencing, and initial phasing. Default parameters were utilized for modeling and quantification in ‘OspreyFit’, including a metabolite fit range of 0.5 to 4.0 ppm, a water fit range of 2.0 to 7.4 ppm, and a knot spacing of 0.4 ppm. The basis set provided for LCModel included simulated metabolite spectra for N-acetylaspartate (NAA), creatine (Cr), choline (Cho), glutamate (Glu), glutamine (Gln), myo-inositol (mI), γ-aminobutyric acid (GABA), and others, generated with density‐matrix simulations at 7T incorporating sequence‐specific TE and mixing time. In addition to the basis set, default macromolecular and lipid components provided by Osprey were fitted to each spectrum. Signal-to-noise (SNR), full-width half-maximum (FWHM) and fractional volume of gray matter, white matter and CSF were calculated in Osprey. Each participant’s ^1^HMRS voxel was then co-registered to their T_1_-weighted MP2RAGE. Example spectra and voxel overlap across participants are shown in Fig. 1. Tissue- and relaxation-corrected metabolite molal concentrations were then estimated in ‘OspreyQuantify’ according to the Gasparovic method [94]. Only tissue-corrected metabolite estimates passing quality control (Cramér–Rao lower‐bound (CRLB) ≤ 20%) were included in subsequent analyses.

### Statistical analysis

Group differences in cannabis use rates were assessed using independent-samples t-tests and chi-squared tests, as appropriate. To examine the effects of ^1^HMRS glutamate, cannabis use, and their interaction on clinical symptom dimensions, we conducted a series of linear regression models. The primary outcome measures of interest were those associated with psychosis, namely positive and negative symptoms, modeling each as a continuous outcome. All linear models controlled for sex and age at scan. Cannabis use was coded dichotomously (0 = non-user, 1 = user), and glutamate was entered as a continuous variable. Interaction terms between glutamate and cannabis user status were included to assess modulation effects. Significance was assessed at *p* < 0.05 (two-tailed), with trend-level effects noted at *p* < 0.10. Residuals were visually inspected for normality and homoscedasticity. Exploratory analyses evaluated other symptom domains (depression, mania, and ADHD, as well as categorical diagnostic group (TD, CHR, PSY; See Supplemental Results)). Demographic data were compared across diagnostic groups (e.g., diagnosis, cannabis user status) using t-tests and chi-square as appropriate. Rates of cannabis use and urine drug screen data were compared across all three diagnostic groups using an omnibus Chi-square test, with planned post-hoc pairwise comparisons using simple slopes (‘sim_slopes’) and least squares means approach (‘lsmeans’) using the ‘interactions’ [95] and ‘lsmeans’ [96] libraries in R (V4.3.3), respectively. Robust regression was completed using ‘rlm’ in the MASS package [97].

## Results

### Self-reported cannabis use rates converge with results of urine toxicology screening

**Fig. 2:**
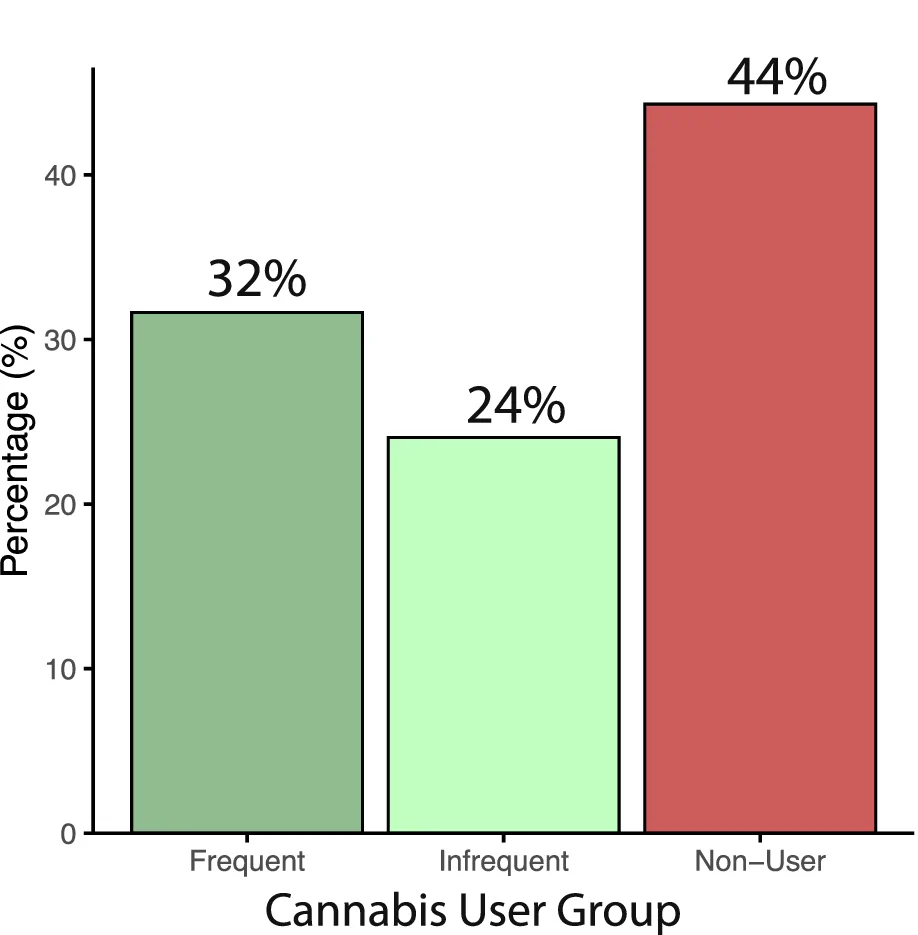
Frequency of self-reported cannabis use in the transdiagnostic sample that underwent 7T MRI. Bars show the percentage of participants (*N* = 79) in each cannabis use category, color-coded by group: frequent users (32%, *n* = 25), infrequent users (24%, *n* = 19), and non-users (44%, *n* = 35). Frequent and infrequent users together comprise the 56% (*n* = 44) of the sample reporting lifetime cannabis use. Data labels above each bar denote the percentage of the total sample.

**Fig. 3:**
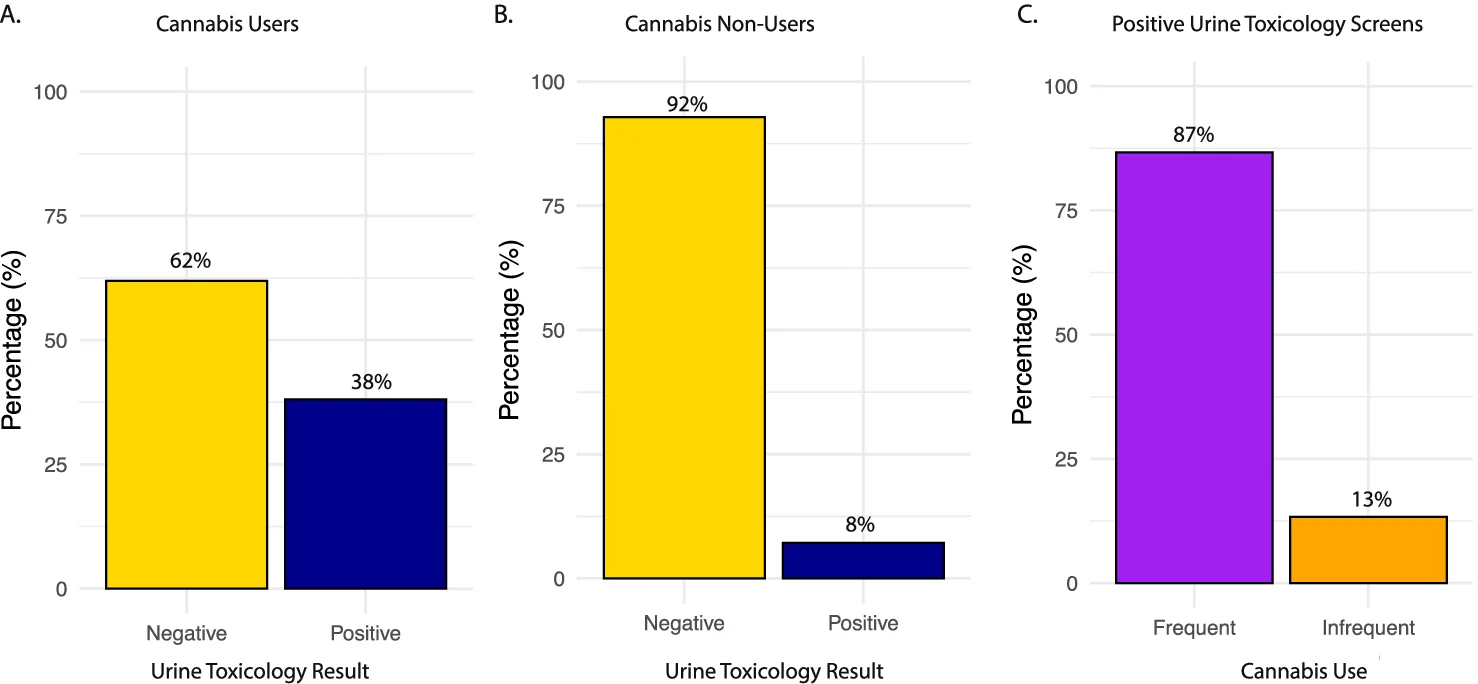
Comparison of self-reported use and urine toxicology screening. **A**. Many, but not a majority, of self-reported cannabis users tested positive on a urine toxicology screen. Two cannabis users did not complete urine toxicology screening. **B**. The vast majority of self-reported cannabis non-users tested negative on a urine toxicology screen. Seven cannabis non-users did not complete urine toxicology screening. **C**. Self-reported frequent cannabis users were more likely to test positive on a urine toxicology screen as compared to infrequent users.

Across the sample, 56% (*n* = 44) self-reported lifetime cannabis use, including 32% (*n* = 25) frequent users and 24% (*n* = 19) infrequent users, while 44% (*n* = 35) were cannabis non-users (Fig. 2). To assess validity of self-reported cannabis use, we compared responses on cannabis use self-report assessments to results from urine toxicology across all individuals. 89% (70/79) of individuals had a valid urine toxicology screen, and self-reported use was significantly related to toxicology results, χ²(1) = 7.62, *p* < 0.01. The vast majority (92%) of cannabis non-users tested negative on the urine toxicology screen (Fig. 3A), while self-reported cannabis users were more likely to test positive on the urine toxicology screen (Fig. 3B), but overall rates of positive screens were low (38%), which is not unexpected given the typical window of cannabis metabolite detection. Indeed, self-reported frequent users (87%) were more likely to test positive on urine toxicology than infrequent users (13%; Fig. 3C). Overall, results indicate good concordance between self-report and biological verification of cannabis use. Note that two individuals who denied cannabis use tested positive on urine toxicology; these individuals were considered infrequent cannabis users in subsequent analyses. Prevalence rates (yes/no) of current nicotine and alcohol use are reported in Supplementary Tables 3 and 4.

### Cannabis use is higher in psychosis patients

76% of PSY participants, 48% of CHR participants, and 45% of TD participants reported cannabis use (Supplementary Fig. 1). Comparison across all three groups was not significant (χ²(2) = 5.51, *p* = 0.06), but in planned pairwise comparisons, cannabis use was significantly more prevalent in PSY compared to both TD (χ²(1) = 4.94, *p* = 0.03; Yates-corrected *p* = 0.05) and CHR (χ²(1) = 3.88, *p* = 0.049; Yates-corrected *p* = 0.09). CHR and TD did not differ in cannabis use (χ²(1) = 0.05, *p* = 0.82, Yates-corrected *p* = 0.99). Notably, CHR (33%) and PSY (32%) showed similar rates of frequent use (3–4 times a week or daily; Supplementary Fig. 2).

### Glutamate and cannabis use are associated with dimensional clinical symptoms

**Fig. 4:**
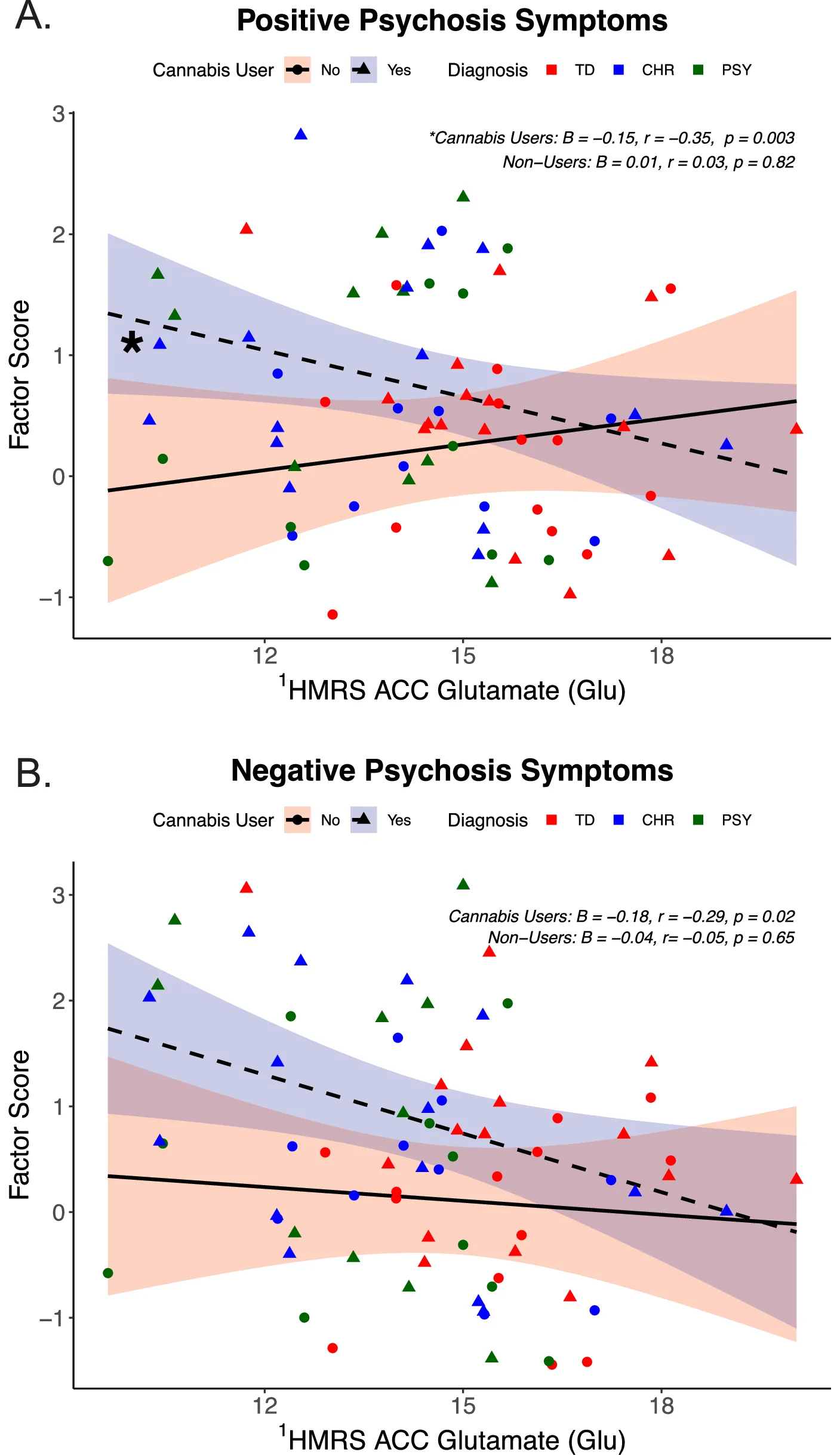
Glutamate × cannabis interactions predicting dimensional psychosis symptoms. Scatterplots show associations between ^1^HMRS anterior cingulate cortex (ACC) glutamate (Glu) and positive (**A**) and negative (**B**) psychosis factor scores, stratified by cannabis use status (yes/no). Points are colored by diagnostic group (red = TD, blue = CHR, green = PSY) and shaped by cannabis use status (circles = non-users, triangles = users). Solid and dashed lines represent regression fits for non-users and users, respectively, with shaded bands indicating 95% confidence intervals. Unstandardized regression coefficients (**B**), partial correlations (*r*), and *p*-values for each simple slope are displayed. An asterisk (*) indicates a significant Glu × cannabis interaction.

We examined the relationship of HMRS glutamate levels, cannabis use, and their interaction with dimensional clinical symptoms across the entire sample. A significant Glu × cannabis interaction was observed for positive psychosis symptoms (*F*(1,68) = 4.33, *p* = 0.04, Cohen’s f² = 0.05), indicating that the relationship between glutamate and positive symptom severity differed by cannabis use status (Fig. 4A). Using a simple slopes follow-up analysis, lower glutamate was significantly associated with greater positive symptom severity in cannabis users (*β* = –0.15, *SE* = 0.05, *t*(68) = –3.11, *p* = 0.003), but this association was not significant in non-users (*β* = 0.01, *SE* = 0.06, *t*(68) = 0.23, *p* = 0.82). These findings suggest that glutamate levels are differentially linked to positive psychosis symptoms depending on cannabis use history, with effects most pronounced among cannabis users. Additionally, both lower glutamate (*p* = 0.012) and higher cannabis use (*p* < 0.001) independently predicted greater positive symptoms. For negative psychosis symptoms (Fig. 4B), lower glutamate (*p* = 0.01) and higher cannabis use (*p* = 0.02) were also significant predictors, but there was no interaction, suggesting additive effects of elevated glutamate and cannabis exposure. Examination of influential observations indicated no undue leverage on results (Supplemental Results).

A series of sensitivity analyses was completed. First, analyses comparing glutamate levels by psychosis diagnostic group showed similar results, including when stratified by cannabis use status (Supplementary Fig. 4 & 5). Next, to assess potential confounding by antipsychotic medication, we compared antipsychotic use rates between cannabis users (14%) and non-users (11%), and chlorpromazine equivalent (CPZE) doses among medicated participants (cannabis users: 206 ± 213 mg, non-users: 87 ± 24 mg; Wilcoxon *p* = 0.51). No statistically significant differences were detected in antipsychotic use or CPZE in cannabis users and non-users. The primary model was re-estimated, including antipsychotic medication status as a covariate; the Glu × cannabis interaction remained significant (F(1,67) = 4.13, *p* = 0.04). Neither the Glu × Antipsychotic interaction (*p* = 0.82) nor the three-way interaction (*p* = 0.96) was significant, indicating that the interaction persisted after adjustment for medication status*.* Similarly, accounting for urine toxicology status and nicotine use did not significantly alter these findings (Supplemental Results).

**Fig. 5:**
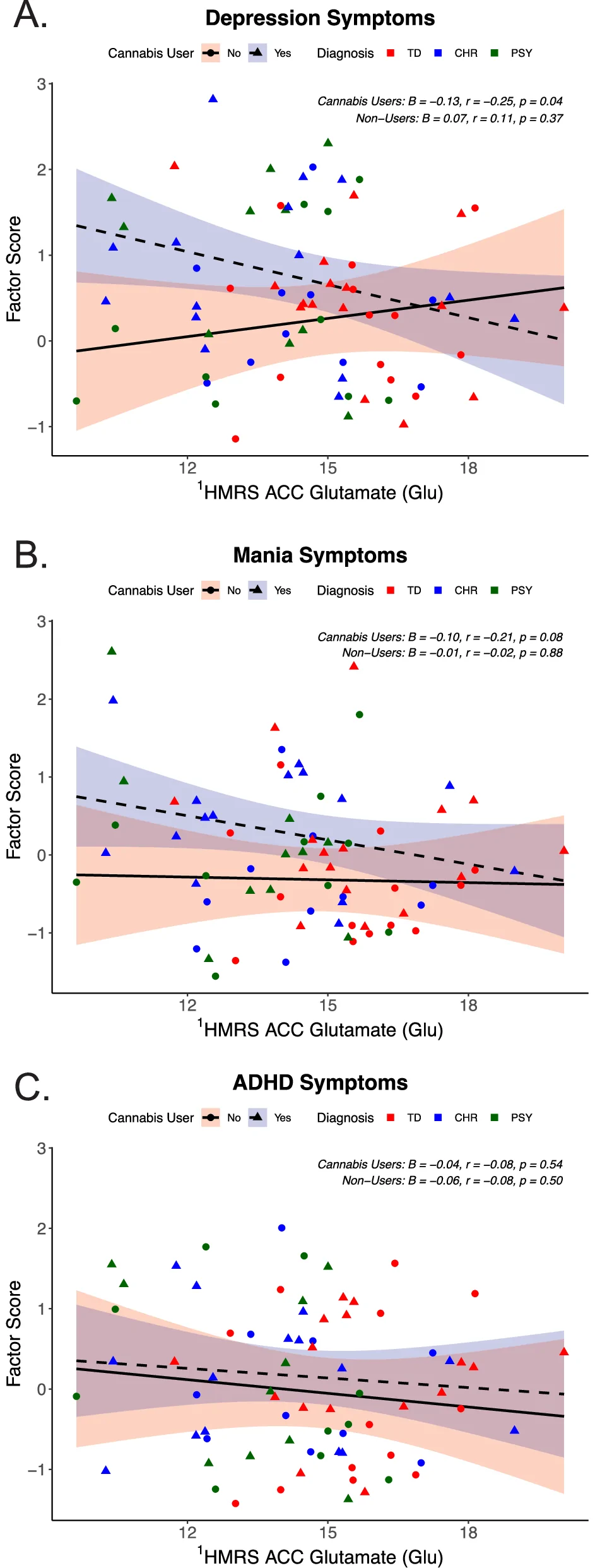
Exploratory glutamate × cannabis associations with depression, mania, and ADHD symptoms. Scatterplots show associations between ^1^HMRS anterior cingulate cortex (ACC) glutamate (Glu) and depression (**A**), mania (**B**), and ADHD (**C**) factor scores, stratified by cannabis use status (yes/no). Points are colored by diagnostic group (red = TD, blue = CHR, green = PSY) and shaped by cannabis use status (circles = non-users, triangles = users). Solid and dashed lines represent regression fits for non-users and users, respectively, with shaded bands indicating 95% confidence intervals. Unstandardized regression coefficients (**B**), partial correlations (*r*), and *p*-values for each simple slope are displayed.

In exploratory analysis of other dimensional symptoms, cannabis use was associated with greater depressive symptoms severity (*p* = 0.01), and a trend-level interaction with glutamate (*p* = 0.05), which suggested a potential cannabis-dependent relationship between glutamate levels and depressive symptoms (Fig. 5A). There was no main effect of glutamate (*p* = 0.22). A similar pattern emerged for manic symptoms, where cannabis use again predicted greater symptom burden (*p* = 0.01), while glutamate showed a marginal association (*p* = 0.09), but with no significant interaction (Fig. 5B). In contrast, ADHD symptoms were not significantly associated with either glutamate or cannabis use (Fig. 5C). Higher glutamate levels were also associated with overall functioning as measured by the GAF (F(1,66) = 4.50, *p* = 0.03), with cannabis non-users showing trend-level (*p* = 0.06) higher GAF scores than cannabis users. Within the cannabis user group, there were no associations between glutamate level and age of onset, length of use, or paranoia scores on the CEQ. There was a trend association of lower levels of glutamate, with higher reported CEQ euphoric scores (*p* = 0.07). Analyses stratified by clinical diagnosis showed similar trends (Supplemental Results).

Concentrations, CRLBs, spectral quality metrics (SNR, FWHM), and voxel tissue fractions for all quantified metabolites are reported by diagnostic group and cannabis user status in Supplementary Tables 5–8. Of these, only glutamate levels differed across groups.

## Discussion

We combined high-resolution 7T ^1^HMRS with detailed cannabis use assessments to examine glutamatergic alterations in a transdiagnostic sample of patients with psychosis, clinical high risk for psychosis, and typically developing youth. Cannabis use was common overall but most prevalent among individuals with psychosis. Self-reported use showed good concordance with urine toxicology results overall, with strongest concordance among frequent users, supporting the validity of self-report in this cohort while highlighting heterogeneity in recency of use. As expected, psychosis participants exhibited elevated clinical symptomatology and lower global functioning compared to CHR and TD groups. Collectively, the results indicate that cannabis use is robustly associated with elevated symptoms across multiple clinical domains—particularly psychosis, but also depression and mania—and that glutamate levels contribute independently to symptom severity, with interaction effects most apparent for positive symptoms, and potentially depressive symptoms. These findings suggest that glutamatergic dysregulation may play a role in symptom expression among cannabis users, especially for the positive symptom dimension of psychosis.

A novel finding of this study was that lower ACC glutamate levels were significantly associated with high positive psychosis symptoms only among cannabis users, suggesting that cannabis use modulates the glutamate–psychosis relationship. A plausible neurobiological mechanism underlying the observed interaction involves CB1 receptor-mediated modulation of glutamatergic signaling in the ACC. THC, the primary psychoactive constituent of cannabis, acts as a partial agonist at CB1 receptors, which are densely expressed on presynaptic glutamatergic terminals in prefrontal cortical regions [98]. Activation of these receptors suppresses glutamate release [65], which may compound illness-related glutamatergic hypofunction in the ACC. The resulting impairment of ACC excitatory neurotransmission could disrupt error monitoring and salience processing, promoting aberrant salience attribution, a mechanism proposed to underlie the emergence of positive psychotic symptoms [99]. This pattern is consistent with evidence that cannabis can disrupt glutamatergic signaling in CB1 receptor–rich cortical regions, potentially leading to maladaptive reductions in excitatory tone or altered glutamate homeostasis in circuits already vulnerable to dysregulation in psychosis [100]. Such cannabis-related alterations could diminish glutamatergic efficiency, thereby linking lower glutamate levels to greater positive symptom expression among users. These results raise the possibility that ACC glutamate could serve as a biomarker of vulnerability among cannabis-using individuals on the psychosis spectrum, which could inform risk stratification and targeted interventions.

Importantly, glutamate was also independently associated with negative symptoms and global functioning across groups, and cannabis use predicted worse outcomes on these measures, indicating broader relevance of glutamatergic dysfunction beyond cannabis-exposed individuals. These findings should be interpreted in the context of substantial heterogeneity in the prior ^1^HMRS glutamate literature in psychosis discussed above, including differences in ROI placement, quantification approach (Glu vs. Glx), medication status, and clinical composition of study samples. Several of these factors are relevant to the current study. By using ultra-high field 7T ^1^HMRS, we benefit from improved spectral separation of Glu from Gln and enhanced quantification precision relative to 3T approaches [48, 49], allowing us to measure Glu rather than the composite Glx. This is a consequential advance given that Glu and Gln (or Glx) often show divergent patterns across diagnostic groups. Glu tends to be lower in psychosis, whereas Gln and Glx are frequently higher. By isolating Glu, we reduce a key source of confounding that has contributed to mixed findings in the 3T literature.

Regarding medication effects, our sample included both medicated and unmedicated individuals. While antipsychotics may help normalize Glu levels both peripherally and centrally [45, 62, 101], prior work from our group found no evidence that medications significantly affected 7T GluCEST contrast in schizophrenia or CHR [22]. Nonetheless, medication status remains a potential confound, and future studies with larger samples should continue to examine medication effects systematically. Our transdiagnostic, dimensional approach also addresses inconsistency related to patient population differences, as categorical diagnostic groupings can obscure meaningful variation in glutamate–symptom relationships that span clinical boundaries. Additionally, by systematically assessing cannabis use, a factor rarely controlled for in prior spectroscopy studies of psychosis, our results suggest that cannabis use patterns may partially account for some heterogeneity observed in previous glutamate findings. Indeed, the interaction between cannabis use and glutamate in predicting positive symptoms observed here would not be detected in studies that do not assess cannabis use, potentially contributing to null or inconsistent results in the existing literature. Given prior reports of regionally specific glutamate reductions in early and chronic psychosis [22, 23, 100], the additive and interactive effects of cannabis use observed here may reflect either direct neurochemical consequences of cannabis, shared neurodevelopmental risk factors, or a combination of both. These findings support a mechanistic framework in which cannabis use interacts with regional glutamatergic vulnerability to influence the severity and profile of psychopathology. Future longitudinal and ultra-high field imaging studies will be critical to clarify causal pathways linking cannabis use, glutamate dysregulation, and clinical outcomes, to determine whether glutamate-targeted pharmacological or behavioral interventions may be particularly beneficial for cannabis-using individuals with psychosis, and to incorporate detailed cannabis assessment as standard practice when measuring brain glutamate in psychosis.

While these findings could be consistent with the interpretation that cannabis use contributes to maladaptive reductions in ACC glutamate, our cross-sectional design constrains causal inference, and several alternative explanations warrant consideration. One possibility is that a biologically distinct subgroup of individuals with inherently lower glutamatergic tone in the ACC may be more vulnerable to both psychotic symptom expression and cannabis use. In this framework, lower baseline glutamate could contribute to dysphoria, anhedonia, or subthreshold psychotic experiences, consistent with evidence that NMDA receptor hypofunction produces the full spectrum of symptoms [27, 102] that motivate cannabis use as a form of self-medication. This self-medication model is consistent with evidence that individuals in psychosis-spectrum populations most commonly report using cannabis to alleviate negative affect, anhedonia, and distress associated with psychotic-like experiences [103–105]. It is also possible that both lower glutamate and cannabis use reflect shared neurodevelopmental genetic or environmental risk factors, rather than one causing the other.

Moreover, these findings can be positioned within the broader glutamate hypofunction hypothesis of schizophrenia, which posits that reduced NMDA receptor-mediated glutamatergic signaling contributes to both positive and negative symptoms through downstream effects on cortical circuit function and mesolimbic dopamine regulation [106–108]. In this context, the interaction between lower ACC glutamate and cannabis use observed here may reflect a convergence of two sources of glutamatergic compromise: one arising from illness-related pathophysiology and another from CB1R-mediated suppression of glutamate release. Chronic, heavy use may produce sustained downregulation of glutamatergic signaling through tonic CB1R activation and desensitization [18, 109], whereas more acute or intermittent exposure may elicit transient glutamate surges that interact differently with underlying vulnerability [18, 64]. Our self-report and urine toxicology data capture patterns of use but cannot resolve the temporal dynamics of cannabis–glutamate interactions, and future longitudinal studies incorporating repeated ^1^HMRS acquisitions will be critical for distinguishing these possibilities.

We also found that cannabis use was associated with greater severity of depression and mania symptoms across the whole sample, highlighting the transdiagnostic clinical relevance of cannabis use. This is consistent with 1) meta-analytic evidence that heavy cannabis use increases risk for depressive disorders [110]; and 2) epidemiological findings of high comorbidity between cannabis use and mood disorders [111, 112]. The present associations may reflect overlapping neurobiological vulnerabilities—such as altered serotonergic and endocannabinoid signaling—that predispose individuals to both cannabis use and mood dysregulation, or the downstream effects of cannabis-related perturbations in neurotransmitter systems on affective processing.

Self-reported cannabis use showed good overall concordance with urine toxicology results, supporting the validity of self-report in assessing cannabis exposure in psychiatric populations. Across the sample, 92% of individuals denying cannabis use tested negative on urine toxicology, and frequent users were far more likely to test positive than infrequent users (87 vs. 13%), though only 38% of all self-reported users screened positive, reflecting heterogeneity in recency of use. This pattern replicates prior work showing that urine toxicology has high specificity and negative predictive value for cannabis [94, 113] and that concordance is often strongest among individuals with frequent use [114]. Results of exploratory analyses incorporating urine toxicology data suggested that the glutamate–positive symptom association was not substantially influenced by the heterogeneity of recent cannabis exposure within the user group. Thus, the pattern of results likely reflects associations between sustained cannabis use and glutamatergic function, rather than acute pharmacological effects of recent THC exposure. Nonetheless, some discrepancies were observed (e.g., overall low rates of positive screens among self-reported users), underscoring important limitations of toxicology measures. Detection windows for urine THC metabolites are finite and may under-detect use in infrequent users [115], while contextual factors, stigma, and recall error could bias self-reports [116, 117]. These findings highlight the utility of pairing structured self-report cannabis use measures with biological verification, particularly in research on high-risk groups such as individuals with psychosis, where cannabis use is prevalent and clinically relevant [118]. Combining approaches may increase confidence in exposure classification while also capturing use patterns and frequency not discernible from toxicology alone.

Notwithstanding the strengths of combining ultra–high field 7T ^1^HMRS, biological and self-report measures of cannabis use, and dimensional clinical assessment across a transdiagnostic sample, several limitations should be considered. Notably, the cross-sectional design limits causal inference about the relationships among glutamate levels, cannabis use, and psychopathology symptom expression. Longitudinal studies are needed to determine whether cannabis-related glutamatergic alterations precede or follow symptom exacerbation. Although self-reported cannabis use showed strong concordance with urine toxicology results, the binary nature and limited detection window of the urine screen may underestimate recent use, particularly in infrequent users. While the inclusion of clinical high-risk, psychosis-spectrum, and typically developing individuals enhances generalizability, the modest sample size, particularly within diagnostic subgroups, may limit statistical power to detect more nuanced interaction effects or subgroup-specific associations. ^1^HMRS glutamate measures remain unable to fully disentangle Glu from Gln and do not reflect synaptic neurotransmission directly. Additionally, use of a single-region ¹HMRS acquisition limits the ability to characterize broader patterns of glutamatergic alteration across the brain, and future multi-voxel, whole-brain spectroscopic imaging or GluCEST studies will be needed to determine whether the observed associations extend to other regions implicated in psychosis and cannabis use. Antipsychotic use rates and doses were comparable between cannabis users and non-users and the Glu × cannabis interaction remained significant when medication status was covaried. However, the small number of medicated participants limits power to detect subtle medication effects, and future studies with larger samples will be important to fully disentangle these factors.

In conclusion, these findings suggest that cannabis use may interact with psychosis-related vulnerability to accentuate glutamatergic dysfunction, and that this neurochemical alteration may contribute to symptom expression, especially in the domain of positive symptoms. Understanding this relationship is critical to elucidate neurobiological substrates underlying both acute and chronic cannabis effects on mental health.

## Availability of data and materials

The data generated and/or analyzed during the current study is available. Please contact Dr. David Roalf with questions and considerations for data sharing upon reasonable requests. Analysis code is available via github repository and shared upon request.

## Supplementary information

Supplemental Material
