# Supplementary material for: Cannabis use and glutamate across the psychosis spectrum: in vivo evidence from 7T proton magnetic resonance spectroscopy
Source: Mol Psychiatry. 2026 Jun 27;31(11):6360–71. doi: 10.1038/s41380-026-03705-1 (PMC13668679; doi:10.1038/s41380-026-03705-1)
Supplement: Supplementary file 1 [file 41380_2026_3705_MOESM1_ESM.docx]

Supplemental Material for

Cannabis Use and Glutamate across the Psychosis Spectrum: In Vivo Evidence from 7T Proton Magnetic Resonance Spectroscopy

David R. Roalf^1,2^, Tyler M. Moore^1,2^, Jacquelyn Stifelman^1^, Maggie K. Pecsok^1^, Ally Atkins^1^, Erica B. Baller^1^, Monica E. Calkins^1,2^, Mariella De Biasi^1,3,4^, Christian G. Kohler^1,2^, Christina Mastracchio^1^, Arianna Mordy^5^, Heather Robinson^6^, Ravinder Reddy^7^, Ravi Prakash Reddy Nanga^7^, Kosha Ruparel^1,2^, Sage Rush-Goebel^1,2^, Daniel H. Wolf^1,2^, Ruben C. Gur^1,2^, Raquel E. Gur^1,2^ & J. Cobb Scott^1,8^

^1^Department of Psychiatry, University of Pennsylvania Perelman School of Medicine, Philadelphia, PA

^2^Lifespan Brain Institute, Department of Child and Adolescent Psychiatry and Behavioral Sciences, Children’s Hospital of Philadelphia, USA  ^3^Pharmacology Graduate Group, Perelman School of Medicine, University of Pennsylvania, Philadelphia, PA, United States

^4^Department of Neuroscience, Perelman School of Medicine, University of Pennsylvania, Philadelphia, PA, United States

^5^Department of Psychology, University of California Los Angeles, Los Angeles, CA, United States

^6^Department of Psychological Sciences, University of Connecticut, Storrs CT, United States

^7^Center for Advanced Metabolic Imaging in Precision Medicine (CAMIPM), Perelman School of Medicine, University of Pennsylvania, Philadelphia, PA, USA

^8^VISN4 Mental Illness Research, Education, and Clinical Center at the Corporal Michael J. Crescenz VA Medical Center, Philadelphia, Pennsylvania

**Supplemental Methods**

**Clinical Psychopathology Assessment**

To facilitate efficient data collection, the clinical assessment instruments were integrated into one administrative tool, the Computer-Assisted Psychopathology Assessment (CAPA).^74^ Interviewers underwent formal training including lectures and mock interviews, followed by supervised administration and performance evaluation until competency was established^74^. For minors, collateral information was provided by an independent interview with a parent or guardian. All information was integrated to yield consensus diagnoses and clinical symptom ratings at a clinical case conference led by at least two doctoral-level clinicians.

**Method for Estimation of Correlated Traits Clinical Factor Scores.**

Estimation of clinical factor scores was done using an exploratory structural equation model (ESEM) with geomin rotation and clustering by subject due to the repeated-measures nature of the data. The number of factors to extract was determined by interpretability and visual examination of the scree plot (based on polychoric correlations), as well as the following empirical methods: minimum average partial method^1^, parallel analysis with Glorfeld correction^2^, and the Kaiser rule (# factors = eigenvalues > 1.0). The ESEM was estimated using the mean- and variance-adjusted weighted least-squares (wlsmv) estimator^3, 4^ in Mplus v8.4^5^. Model fit was assessed using the acceptability cutoffs suggested by Hu and Bentler^6^ via the root mean-square error of approximation (RMSEA; acceptable = 0.06), comparative fit index (CFI; acceptable = 0.95), Tucker-Lewis index (TLI; acceptable = 0.95), and standardized root mean-square residual (SRMR; acceptable = 0.08). This model was used to calculate factor scores using the maximum a posteriori (MAP) method. Additional details are provided in the Supplement.

Results from the empirical tests for the number of factors were mixed. Parallel analysis suggested eleven factors or eight principal components; the MAP method suggested five factors; the Kaiser rule suggested eight factors; and the elbow of the scree plot suggested four factors. Based on interpretability and the tendency of empirical methods to over-extract, we settled on five factors, expecting approximately nine items per factor. Fit of the model was acceptable (CFI = 0.96; TLI = 0.95; RMSEA = 0.032 ± 0.001; SRMR = 0.036), and Supplemental Table 1 shows the resulting factor pattern (loading) matrix. Factor 1 (ADHD) comprises all ADHD items and the SIPS Disorganized item (SIPD013) “Trouble with Focus and Attention”; Factor 2 (Psychosis Positive Symptoms) comprises all PRIME screen items; Factor 3 (Mania) comprises all mania items; Factor 4 (Depression) comprises all depression items; and Factor 5 (Psychosis Negative Symptoms) comprises all SIPS Negative items and two Positive items (SIPP090a and SIPP092) “Do people ever tell you that they can't understand you?” and “Disorganized Communication Severity Scale”. Inter-factor correlations (phi) were moderate (mean = 0.40), suggesting a general “p” factor underlying all items^7^.
**Supplemental Table 1. Factor Loadings for Correlated Traits Model**

| **Item** | **F1 (ADHD)** | **F2 (Psychosis Positive)** | **F3 (Mania)** | **F4 (Depression)** | **F5 (Psychosis Negative)** |
| --- | --- | --- | --- | --- | --- |
| ADD011 | 0.875 | -0.005 | 0.016 | -0.018 | 0.082 |
| SIPD013 | 0.799 | -0.014 | -0.004 | -0.084 | 0.181 |
| ADD022 | 0.795 | 0.107 | 0.041 | 0.010 | -0.162 |
| ADD016 | 0.781 | 0.064 | 0.081 | -0.021 | 0.022 |
| ADD021 | 0.776 | 0.078 | 0.056 | 0.027 | -0.167 |
| ADD014 | 0.732 | 0.044 | 0.033 | 0.018 | -0.012 |
| ADD020 | 0.700 | 0.058 | 0.188 | -0.019 | -0.082 |
| ADD015 | 0.696 | 0.067 | -0.008 | 0.069 | 0.050 |
| ADD013 | 0.667 | -0.095 | 0.117 | 0.119 | 0.066 |
| ADD019 | 0.443 | -0.150 | -0.087 | 0.075 | 0.081 |
| ADD018 | 0.375 | -0.020 | -0.155 | 0.008 | 0.090 |
| ADD017 | 0.332 | -0.142 | -0.075 | 0.091 | 0.091 |
| SIP010 | 0.002 | 0.826 | -0.012 | -0.129 | -0.020 |
| SIP005 | 0.025 | 0.782 | -0.031 | 0.020 | 0.029 |
| SIP004 | 0.002 | 0.782 | -0.040 | -0.109 | -0.007 |
| SIP008 | 0.009 | 0.780 | 0.005 | -0.036 | -0.052 |
| SIP007 | 0.017 | 0.775 | 0.046 | 0.025 | 0.001 |
| SIP011 | 0.010 | 0.760 | -0.005 | 0.080 | 0.073 |
| SIP013 | -0.026 | 0.754 | 0.082 | 0.008 | -0.011 |
| SIP006 | -0.028 | 0.737 | 0.034 | 0.016 | 0.003 |
| SIP012 | 0.025 | 0.713 | 0.064 | 0.033 | 0.044 |
| SIP003 | -0.013 | 0.671 | 0.074 | 0.072 | 0.060 |
| SIP009 | 0.051 | 0.652 | -0.003 | 0.108 | 0.051 |
| SIP014 | 0.030 | 0.631 | -0.023 | 0.191 | 0.161 |
| MAN002 | 0.061 | -0.004 | 0.900 | -0.036 | 0.007 |
| MAN001 | 0.088 | -0.015 | 0.851 | -0.001 | 0.009 |
| MAN005 | -0.058 | 0.003 | 0.840 | 0.084 | 0.038 |
| MAN003 | 0.023 | 0.026 | 0.811 | -0.005 | 0.066 |
| MAN004 | 0.082 | 0.023 | 0.799 | 0.050 | 0.016 |
| MAN006 | -0.030 | 0.056 | 0.788 | 0.019 | -0.002 |
| MAN007 | 0.013 | -0.055 | 0.643 | 0.375 | 0.016 |
| DEP001 | 0.015 | 0.022 | 0.041 | 0.850 | 0.061 |
| DEP002 | 0.006 | 0.046 | 0.113 | 0.792 | -0.046 |
| DEP149 | 0.103 | 0.102 | -0.071 | 0.662 | 0.115 |
| DEP004 | 0.098 | 0.001 | 0.300 | 0.653 | -0.003 |
| DEP006 | 0.044 | 0.089 | 0.236 | 0.619 | 0.021 |
| DEP148 | 0.081 | 0.137 | 0.010 | 0.515 | 0.091 |
| SIPN020 | -0.182 | 0.017 | 0.116 | 0.193 | 0.774 |
| SIPN011 | 0.237 | -0.052 | -0.156 | 0.021 | 0.770 |
| SIPP092 | 0.104 | 0.090 | 0.095 | -0.355 | 0.764 |
| SIPN035 | 0.230 | -0.037 | -0.147 | 0.027 | 0.666 |
| SIPP090A | 0.043 | 0.042 | 0.340 | -0.440 | 0.643 |
| SIPN014 | -0.043 | 0.083 | -0.106 | -0.048 | 0.628 |
| SIPN013A | -0.179 | 0.023 | 0.192 | 0.152 | 0.616 |
| SIPN009 | 0.168 | -0.088 | -0.015 | 0.151 | 0.544 |
| SIPN019 | -0.139 | 0.070 | 0.168 | 0.344 | 0.477 |
| **Inter-Factor Correlations (Phi)** | | | | | |
|  | **F1** | **F2** | **F3** | **F4** | **F5** |
| F1 | 1.000 | 0.314 | 0.390 | 0.247 | 0.414 |
| F2 | 0.314 | 1.000 | 0.520 | 0.341 | 0.465 |
| F3 | 0.390 | 0.520 | 1.000 | 0.359 | 0.377 |
| F4 | 0.247 | 0.341 | 0.359 | 1.000 | 0.553 |
| F5 | 0.414 | 0.465 | 0.377 | 0.553 | 1.000 |

Note. ADHD = attention deficit hyperactivity disorder; F = factor.

**^1^HMRS acquisition**

^1^HMRS data were collected with a short echo time custom-modified Point RESolved Spectroscopy (PRESS) sequence with the following acquisition parameters: averages = 8 (water reference) / 64 (water suppressed), TE = 23 ms and TR = 3000 ms. Acquisition time was 36 seconds for the reference spectrum and 3 minutes 24 seconds for the suppressed spectrum. The water reference scan was used to compute the absolute concentration of brain neurochemicals, including absolute glutamate concentration. Water suppression was achieved with variable power RF pulses with optimized relaxation delays (VAPOR) prior to the acquisition. VAPOR implements seven frequency selective RF pulses to reduce sensitivity to variations in the B1 field. Pulse amplitudes were calibrated before measurement to reduce B1 inhomogeneity by acquiring six localized STimulated Echo Acquisition Mode (STEAM) water spectra at fixed reference voltage and varying flip angle from 0º to 180º with a step size of 30º. The vendor-provided FASTESTMAP shimming method (from vendor Siemens Medical Solutions, Erlangen, Germany) was employed to minimize spectral linewidths (< 30 Hz) of water signal.

**Supplemental Results**

**Self-reported cannabis use stratified by diagnostic clinical group.**

**Supplemental Figure 1. Self-reported cannabis use rates stratified by clinical diagnosis.**

**Supplemental Figure 2. Self-reported frequency of usage stratified by clinical diagnosis**

**Dimensional clinical factor scores by diagnosis group.**

**Supplemental Table 2. Correlated Traits Clinical Factors Scores by diagnostic and cannabis use.**

|  | **N** | **Positive** | **Negative** | **Depression** | **Mania** | **ADHD** |
| --- | --- | --- | --- | --- | --- | --- |
| **TD** | | | | | | |
| Cannabis User | | | | | | |
| No | 16 | -0.44 ± 0.40 | -0.21 ± 0.72 | 0.00 ±  0.69 | -0.52 ± 0.52 | -0.54 ± 0.62 |
| Yes | 15 | -0.22 ± 0.40 | -0.08 ± 0.68 | 0.12 ±  0.74 | -0.18 ± 0.64 | -0.41 ± 0.57 |
| **CHR** | | | | | | |
| Cannabis User | | | | | | |
| No | 14 | 0.22 ± 0.70 | 0.92 ± 0.80 | 0.29 ±  0.99 | 0.12 ± 0.94 | 0.37 ± 1.03 |
| Yes | 13 | 0.48 ± 0.72 | 1.16 ± 1.13 | 1.05 ±  0.78 | 0.48 ± 1.00 | 0.44 ± 0.86 |
| **PSY** | | | | | | |
| Cannabis User | | | | | | |
| No | 5 | 0.00 ± 0.45 | 1.24 ± 0.51 | 0.69 ±  1.05 | 0.24 ± 1.03 | 1.02 ± 1.04 |
| Yes | 14 | 1.15 ± 0.66 | 1.94 ± 0.91 | 1.34 ±  0.79 | 0.82 ± 0.75 | 0.90 ± 0.66 |

Correlated Traits Clinical Factor scores (mean ± SD) by diagnostic group (TD, CHR, PSY) and cannabis use. TD = Typically Developing; CHR = Clinical High Risk; PSY = Psychosis. ADHD = Attention Deficit Hyperactivity Disorder.

**Robustness of interaction between glutamate and cannabis status on positive psychosis symptoms.**

**Supplemental Figure 3. Cook’s distance plots indicated there were three potential influential outliers**

To evaluate the robustness of the Glu × cannabis interaction for positive symptoms, we conducted several sensitivity analyses. Robust regression yielded a consistent interaction estimate (*β* = -0.14, *p* = 0.06), and a nonparametric bootstrap (5,000 iterations) confirmed that the 95% confidence interval for the interaction term excluded zero (percentile: -0.29 to -0.03; BCa: -0.31 to -0.040). Examination of Cook's distance identified three mildly influential observations (Cook's D = 0.06–0.13), all well below conventional thresholds for concern (Cook's D > 0.50; Supplemental Figure 3). Together, these analyses indicate that the Glu × cannabis interaction is not driven by outliers or influential observations.

**Addressing heterogeneity of self-reported cannabis status and urine toxicology assay.** To examine whether heterogeneity of recent cannabis exposure within the user group influenced our primary findings, we conducted exploratory analyses of positive psychosis symptoms that incorporated urine toxicology results. Among cannabis users only (n = 42), the Glu × urine toxicology interaction was not significant (F(1,33) = 1.47, p = 0.23), indicating that the glutamate–positive symptom relationship did not significantly differ between tox-positive and tox-negative users, though power was limited. In a complementary analysis using a three-level exposure variable (Non-User/Tox-, n = 26; User/Tox-, n = 26; User/Tox+, n = 16), the main effect of glutamate (F(1,56) = 6.23, p = 0.01) and cannabis exposure group (F(2,56) = 5.74, p < 0.01) remained significant, but there was no interaction between glutamate and cannabis exposure group (p = 0.17). Together, these results suggest that the primary findings are not substantially influenced by the heterogeneity of recent cannabis exposure within the user group, though the modest subgroup sizes limit power to detect more nuanced differences.

**Prevalence of alcohol and nicotine use within the sample**

Alcohol and nicotine use data were available for 62 and 73 of 79 participants, respectively. Alcohol and nicotine use did not differ significantly across diagnostic groups (alcohol: PSY 83.3%, PRO/CHR 76.2%, TD/NC 73.9%, p = .76; nicotine: PSY 50.0%, PRO/CHR 36.0%, TD/NC 43.3%, p = .65). However, cannabis users had significantly higher rates of both alcohol use (100.0% vs. 48.1%, p < .001) and nicotine use (68.4% vs. 14.3%, p < .001) compared to non-users.

To evaluate whether nicotine use confounded the glutamate × cannabis interaction, we re-estimated the primary positive psychosis model including nicotine use status as an additional covariate in the subsample with available data (n = 73). The nicotine covariate was non-significant (β=0.04, p = .847), and the interaction estimate was essentially unchanged (β=−0.107, p = .162 vs. β=−0.108, p = .152 without the nicotine covariate in the same subsample). The modest attenuation relative to the full-sample result (β=−0.162, p = .04) likely reflects reduced statistical power from missing nicotine data rather than confounding, as demonstrated by the near-identical estimates with and without the nicotine term in the complete-case subsample.

**Supplemental Table 3. Substance Use Prevalence by Cannabis User Status**

|  |  | THC User | THC Non-User | p-value |
| --- | --- | --- | --- | --- |
| **Alcohol** | User | 35 (100.0%) | 13 (48.1%) | 0.000 |
|  | Non-User | 0 (0.0%) | 14 (51.9%) |  |
|  | Missing | 9 | 8 |  |
|  |  |  |  |  |
| **Nicotine** | User | 26 (68.4%) | 5 (14.3%) | 0.000 |
|  | Non-User | 12 (31.6%) | 30 (85.7%) |  |
|  | Missing | 6 | 0 |  |

Prevalence of alcohol and nicotine use by cannabis user status (THC User vs. THC Non-User). Values represent N (%) based on the earliest available self-report assessment. P-values are from chi-square tests (or Fisher's exact test where expected cell counts were < 5).

Supplemental Table 4. Substance Use Prevalence by Diagnostic Group

|  |  | PSY | CHR | TD | p-value |
| --- | --- | --- | --- | --- | --- |
| **Alcohol** | User | 15 (83.3%) | 16 (76.2%) | 17 (73.9%) | 0.763 |
|  | Non-User | 3 (16.7%) | 5 (23.8%) | 6 (26.1%) |  |
|  | Missing | 3 | 6 | 8 |  |
|  |  |  |  |  |  |
| **Nicotine** | User | 9 (50.0%) | 9 (36.0%) | 13 (43.3%) | 0.652 |
|  | Non-User | 9 (50.0%) | 16 (64.0%) | 17 (56.7%) |  |
|  | Missing | 3 | 2 | 1 |  |

Prevalence of alcohol and nicotine use by diagnostic group (PSY, PRO/CHR, TD/NC). Values represent N (%) based on the earliest available self-report assessment. P-values are from chi-square tests (or Fisher's exact test where expected cell counts were < 5).

**^1^HMRS glutamate levels are lower in PSY who use cannabis**

As a follow-up to the dimensional analyses, we conducted a sensitivity analysis comparing glutamate levels by diagnostic group while adjusting for age and sex. A linear model revealed a significant main effect of diagnosis on ^1^HMRS glutamate levels, *F*(2, 72) = 4.25, *p* = 0.01 (Supplemental Figure 4). Neither age (*p* = 0.10) nor sex (*p* = 0.52) were significant predictors of glutamate level. Post hoc pairwise comparisons showed that participants with psychosis (PSY) had significantly lower glutamate levels compared to TD (p = 0.01), while comparisons between PSY and CHR (p = 0.15) and between CHR and TD (p = 0.47) were not significant (Supplemental Figure 4).

**Supplemental Figure 4. ACC glutamate levels are lower in PSY patients as compared to TD.**

As a follow up to determine whether the effect of diagnosis group on glutamate levels varied by cannabis use, we stratified the analysis by cannabis user status (Yes/No). Among individuals who reported cannabis use, diagnosis group remained a significant predictor of ^1^HMRS glutamate level, *F*(2, 37) = 3.77, *p* = 0.03, while age (*p* = 0.26) and sex (*p* = 0.74) were not significant (Supplemental Figure 5). In contrast, among non-users, there was no significant effect of diagnosis group on ^1^HMRS glutamate level, *F*(2, 22) = 0.02, *p* = 0.98, while age (*p* = 0.52) and sex (*p* = 0.28) were not significant. Exploratory correlations between glutamate levels and clinical symptoms indicated that higher glutamate levels were associated with lower negative SIPS scores, r(73) = -0.33, *p* = 0.003, which was primarily driven by the PSY cannabis users, r(12) = -0.54. Considered together, these findings indicate robust diagnostic group differences in glutamate levels, independent of demographic covariates, and suggest glutamate differences are primarily evident in PSY patients who use cannabis. Finally, and as expected, when stratified by clinical diagnosis, symptoms were significantly higher in PSY as compared to CHR and TD in each SIPS domain (positive, negative, disorganized and general) and GAF scores were lower in PSY as compared to CHR and TD (Supplemental Table 1). Given the small sample size of cannabis non-users in the PSY group, exploratory pairwise comparisons between cannabis users and non-users were conducted within each diagnostic group to assess differences in clinical symptom scores. Among individuals with PSY, cannabis users exhibited trend level higher positive symptoms, *p* = 0.11, disorganized symptoms (*p* = 0.09) and lower GAF scores (*p* = 0.12), compared to PSY non-users. There were no other trend or significant differences in cannabis users as compared to non-users. CEQ summary scores for paranoia and euphoria did not differ by diagnostic group. There were no diagnostic group differences in age at first cannabis use or length of cannabis use.

**Supplemental Figure 5. ACC glutamate is lower in PSY patients who use cannabis as compared to TD who use cannabis. Glutamate levels were similar in non-users.**

Cannabis use was more prevalent among participants with psychosis (PSY) than in either the clinical high-risk (CHR) or typically developing (TD) groups, consistent with extensive epidemiological evidence linking cannabis exposure to both increased risk for and worsened outcomes in psychosis ^8-10^. In the present sample, 76% of PSY participants reported lifetime cannabis use compared to 48% of CHR and 45% of TD individuals, with planned comparisons revealing a significant PSY–TD difference and a trend toward higher rates in PSY than CHR. These findings are consistent with prior studies that show cannabis use is common in psychosis—up to 40% of patients^10^—and may contribute to illness onset and progression through both direct neurobiological effects of Δ9-THC and shared genetic or environmental vulnerability. The elevated use observed here reinforces the importance of routine cannabis screening in psychosis and early intervention services, especially in the context of rising THC potency and cannabis commercialization^9^.

**Heterogeneity of urine toxicology results**

To examine whether heterogeneity of recent cannabis exposure within the user group influenced our primary findings, we conducted exploratory analyses of positive psychosis symptoms, but now incorporating urine toxicology results. Among cannabis users only (n = 42), the Glu × urine toxicology interaction was not significant (F(1,33) = 1.47, p = 0.23), indicating that the glutamate–positive symptom relationship did not significantly differ between tox-positive and tox-negative users, though power was limited. In a complementary analysis using a three-level exposure variable (Non-User/Tox-, n = 26; User/Tox-, n = 26; User/Tox+, n = 16), the main effect of glutamate (F(1,56) = 6.23, p = 0.016) and cannabis exposure group (F(2,56) = 5.74, p = 0.005) remained significant. But there was no interaction between glutamate and cannabis exposure group (p=0.17). Together, these results suggest that the primary findings are not substantially influenced by the heterogeneity of recent cannabis exposure within the user group, though the modest subgroup sizes limit power to detect more nuanced differences.

**^1^HMRS Quality Assurance**

**Supplemental Table 5. Spectral Quality Metrics by Diagnostic Group**

| Measure | TD/ | CHR | PSY | p |
| --- | --- | --- | --- | --- |
| Glutamate CRLB | 2.10 ± 0.30 | 2.27 ± 0.45 | 2.33 ± 0.49 | 0.106 |
| Glutamine CRLB | 8.16 ± 2.07 | 8.00 ± 1.94 | 8.50 ± 2.09 | 0.722 |
| Creatine CRLB | 11.19 ± 6.24 | 11.46 ± 4.17 | 12.56 ± 5.98 | 0.700 |
| GABA CRLB | 9.94 ± 7.30 | 9.12 ± 5.53 | 8.00 ± 1.08 | 0.527 |
| NAA CRLB | 1.39 ± 0.50 | 1.31 ± 0.47 | 1.61 ± 0.50 | 0.126 |
| SNR | 141.21 ± 25.84 | 134.99 ± 24.20 | 125.65 ± 30.29 | 0.130 |
| FWHM | 12.54 ± 2.01 | 13.47 ± 2.93 | 13.24 ± 1.78 | 0.290 |
| Fraction Gray Matter | 0.71 ± 0.10 | 0.69 ± 0.16 | 0.68 ± 0.09 | 0.659 |
| Fraction White Matter | 0.17 ± 0.15 | 0.20 ± 0.21 | 0.24 ± 0.14 | 0.361 |
| Fraction CSF | 0.12 ± 0.09 | 0.12 ± 0.08 | 0.08 ± 0.08 | 0.173 |

Spectral quality metrics by diagnostic group (TD, CHR, PSY), including Cramér-Rao Lower Bounds (CRLBs, %), signal-to-noise ratio (SNR), full width at half maximum (FWHM, Hz), and voxel tissue fractions. P-values are from one-way ANOVAs.

Supplemental Table 6. Spectral Quality Metrics by Cannabis User Status

| Measure | No | Yes | p |
| --- | --- | --- | --- |
| Glutamate CRLB | 2.18 ± 0.39 | 2.24 ± 0.43 | 0.557 |
| Glutamine CRLB | 8.30 ± 2.21 | 8.10 ± 1.86 | 0.667 |
| Creatine CRLB | 11.55 ± 6.38 | 11.67 ± 4.76 | 0.928 |
| GABA CRLB | 8.27 ± 3.15 | 9.90 ± 7.09 | 0.187 |
| NAA CRLB | 1.33 ± 0.48 | 1.48 ± 0.51 | 0.215 |
| SNR | 138.04 ± 28.69 | 132.64 ± 25.40 | 0.388 |
| FWHM | 12.55 ± 1.78 | 13.44 ± 2.65 | 0.084 |
| Fraction Gray Matter | 0.69 ± 0.14 | 0.70 ± 0.11 | 0.615 |
| Fraction White Matter | 0.21 ± 0.19 | 0.18 ± 0.16 | 0.460 |
| Fraction CSF | 0.10 ± 0.08 | 0.12 ± 0.09 | 0.422 |

Spectral quality metrics by cannabis user status (Yes, No), including Cramér-Rao Lower Bounds (CRLBs, %), signal-to-noise ratio (SNR), full width at half maximum (FWHM, Hz), and voxel tissue fractions. P-values are from independent-sample t-tests.

**Neurometabolite concentrations**

**Supplemental Table 7. Neurometabolite Concentrations by Diagnostic Group**

| Measure | TD | CHR | PSY | p |
| --- | --- | --- | --- | --- |
| Glutamate | 15.18 ± 2.14 | 14.42 ± 2.21 | 13.54 ± 1.79 | 0.034 |
| Glutamine | 4.77 ± 1.43 | 4.59 ± 1.20 | 4.28 ± 1.12 | 0.442 |
| Creatine | 3.29 ± 1.03 | 2.82 ± 0.78 | 3.19 ± 0.90 | 0.147 |
| GABA | 4.63 ± 1.43 | 4.62 ± 1.27 | 4.59 ± 0.56 | 0.995 |
| NAA | 13.64 ± 1.76 | 13.24 ± 1.66 | 12.83 ± 1.42 | 0.253 |

Neurometabolite concentrations (mean ± SD) by diagnostic group (TD, CHR, PSY). Concentrations are tissue-corrected and reported in institutional units. P-values are from one-way ANOVAs.

Supplemental Table 8. Neurometabolite Concentrations by Cannabis User Status

| Measure | No | Yes | p |
| --- | --- | --- | --- |
| Glutamate | 14.68 ± 2.00 | 14.40 ± 2.30 | 0.575 |
| Glutamine | 4.54 ± 1.40 | 4.63 ± 1.20 | 0.772 |
| Creatine | 3.13 ± 0.94 | 3.08 ± 0.94 | 0.841 |
| GABA | 4.75 ± 1.08 | 4.51 ± 1.29 | 0.380 |
| NAA | 13.37 ± 1.44 | 13.26 ± 1.83 | 0.769 |

Neurometabolite concentrations (mean ± SD) by cannabis user status (Yes, No). Concentrations are tissue-corrected and reported in institutional units. P-values are from independent-samples t-tests.

References

1.	Velicer WF. Determining the number of components from the matrix of partial correlations. *Psychometrika* 1976; **41**(3)**:** 321–327.

2.	Glorfeld LW. An improvement on Horn's parallel analysis methodology for selecting the correct number of factors to retain. *Educ Psychol Meas* 1995; **55**(3)**:** 377–393.

3.	Beauducel A, Herzberg PY. On the performance of maximum likelihood versus means and variance adjusted weighted least squares estimation in CFA. *Structural Equation Modeling* 2006; **13**(2)**:** 186–203.

4.	Muthén BO. Robust inference using weighted least squares and quadratic estimating equations in latent variable modeling with categorical and continuous outcomes. *Psychometrika* 1997.

5.	Muthén L, Muthén B. Mplus user’s guide. Eight edition. *Muthén & Muthén* 1998; **10**.

6.	Hu Lt, Bentler PM. Cutoff criteria for fit indexes in covariance structure analysis: Conventional criteria versus new alternatives. *Structural equation modeling: a multidisciplinary journal* 1999; **6**(1)**:** 1–55.

7.	Lahey BB, Applegate B, Hakes JK, Zald DH, Hariri AR, Rathouz PJ. Is there a general factor of prevalent psychopathology during adulthood? *J Abnorm Psychol* 2012; **121**(4)**:** 971.

8.	Ganesh S, D’Souza DC. Cannabis and psychosis: recent epidemiological findings continuing the “causality debate”. vol. 179. American Psychiatric Association Washington, DC2022, pp 8–10.

9.	Myran DT, Pugliese M, Roberts RL, Solmi M, Perlman CM, Fiedorowicz J *et al.* Association between non-medical cannabis legalization and emergency department visits for cannabis-induced psychosis. *Mol Psychiatry* 2023; **28**(10)**:** 4251–4260.

10.	Niznikiewicz M, Lin A, DeLisi LE. The Relationship of glutamate signaling to cannabis use and schizophrenia. *Current Opinion in Psychiatry* 2025; **38**(3)**:** 177–181.
